# Downregulation of miR-27a-3p Modulates TGF-β Signaling and Dysregulates Metabolism in Glioblastoma

**DOI:** 10.3390/ijms26178729

**Published:** 2025-09-08

**Authors:** Augusto Ferreira Weber, Juliete Nathali Scholl, Camila Kehl Dias, Vinícius Pierdoná Lima, Tamires de Bona, Renata Marschner, Arieli Cruz de Sousa, Fábio Klamt, Fabrício Figueiró

**Affiliations:** 1 Graduate Program in Biological Sciences: Biochemistry, Institute of Basic Health Sciences, Federal University of Rio Grande do Sul, Porto Alegre 90035-003, RS, Brazil; augustof.weber@gmail.com (A.F.W.); juliete.scholl@gmail.com (J.N.S.); camila.kehldias@gmail.com (C.K.D.); vini.p.lima@gmail.com (V.P.L.); marschnernati@gmail.com (R.M.); arieli.csousa@gmail.com (A.C.d.S.); klamtf@gmail.com (F.K.); 2 Laboratory of Cancer Immunobiochemistry, Department of Biochemistry, Institute of Basic Health Sciences, Federal University of Rio Grande do Sul, Porto Alegre 90035-003, RS, Brazil; tamiresdbona.ufrgs@gmail.com; 3 Department of Biochemistry, Institute of Basic Health Sciences, Federal University of Rio Grande do Sul, Porto Alegre 90035-003, RS, Brazil

**Keywords:** glioblastoma, miR-27a-3p, miR-155-5p, metabolism reprogramming, TGF-β signaling

## Abstract

Several microRNAs (miRNAs) are key influencers of tumor microenvironment (TME) cell plasticity, regulating the progression of various tumor types such as glioblastoma (GBM). Differential expressions of miR-27a-3p and miR-155-5p in GBM cells and biopsies have already been described as markers of tumor subtype and progression. We aimed to evaluate the cellular and molecular impacts of inhibiting these two overexpressed miRNAs in GBM cell lines. A172 cells were transfected with miR-27a-3p and miR-155-5p inhibitors, and the effects on cellular processes and the expression of malignancy-related genes were analyzed by flow cytometry and qPCR, respectively. Thus, several cellular characteristics in A172 cells were modulated; however, only the inhibition of miR-27a-3p resulted in apoptosis, reduced glucose uptake, and a decrease in mitochondrial membrane potential. Both inhibitors modulated metabolic and immunological targets, negatively regulating genes in the glycolysis pathway and modulating other metabolic pathways involving glutamine and fatty acids, for example. Additionally, it modulates the TGF-β pathway, which can influence the GBM microenvironment due to its immunosuppressive role in advanced tumors. miR-27a-3p appears to be a pivotal factor in the functional duality of TGF-β and its interaction with HIF1A in the hypoxic tumor environment, modulating SMAD partners or TGF-β pathway inhibitors. Here, we demonstrate the importance of inhibiting overexpressed miRNAs, particularly miR-27a-3p, in modulating key pathways for tumor cell survival. The results of this work provide new insights into potential targets for immune-metabolic interactions in the TME and their implications for tumorigenesis, shedding light on new therapeutic approaches for GBM.

## 1. Introduction

Glioblastoma (GBM) is a subtype of a central nervous system tumor characterized as grade IV histological malignancy, according to the World Health Organization (WHO) classification [1]. It is considered the most common, aggressive, and deadly form of glioma since the average survival is less than 12 months and accounts for 70% of diffuse glioma diagnoses [2,3,4]. Due to the heterogeneous cellular and molecular characteristics of GBM, the tumor site, and the complicated biology of the disease characterized by frequent relapses and lack of curative therapies, the prognosis of patients with this pathology is dismal [2,5,6]. This scenario results from a poor understanding of GBM molecular pathogenesis. Therefore, it is necessary to elucidate the mechanisms involved in the interaction between tumor cells and the tumor microenvironment (TME) by evaluating potential molecular mechanisms underlying the development and progression of GBM. In this sense, many microRNAs have been studied as potential diagnostic markers and as promising therapeutic targets for glioma management [7].

MicroRNAs (miRNAs) are small non-coding RNAs, approximately 20–25 nucleotides long, that act in the post-transcriptional regulation of gene expression by binding to the 3′ untranslated regions (UTRs) of target mRNAs, resulting in translational repression and/or mRNA degradation [8]. Countless evidence indicates that miRNAs regulate cellular processes related to cancer development [9,10]. Furthermore, based on the miRNA target, aberrant expressions can have either oncogenic (oncomiR) or tumor-suppressive functions in various tumor types, such as GBM [8,11]. Several studies have identified a number of miRNAs with potentially pathogenic functions in gliomas, and the differential expression of these molecules has already been evaluated as possible biomarkers for diagnosis and prognosis in this tumor, even differing according to the degrees of glioma [11,12].

Accumulating evidence suggests that miR-27a-3p and miR-155-5p are upregulated in glioblastoma and can be functionally classified as oncomiRs. Both miRNAs are highly expressed in many tumor types, favoring the processes of tumorigenesis, proliferation, apoptosis, migration, and invasion in GBM [13,14]. Overexpression of miR-27a-3p and miR-155-5p is related to a poor prognosis and resistance to chemotherapy [14,15,16,17]. The differential expression of these miRNAs allows the distinction between glioma subtypes; normally, overexpression favors the highest grades [18,19]. The expression of miR-27a-3p promotes a more aggressive phenotype [20], while miR-155 regulates the Treg/T cell ratio in immune responses [21].

In view of the roles these miRNAs play in tumor progression, direct inhibition of these oncogenic molecules may lead to new therapeutic approaches against GBM. Synthetic oligonucleotides complementary to miRNA sequences can be designed to target and functionally reduce miRNA activity, which can penetrate the blood–brain barrier through association with proteins or via extracellular vesicles [22,23]. Here, we have shown that the inhibition of miR-27a-3p and miR-155-5p interfered with nutrient uptake and expression of metabolism genes and modulated pathways involved in tumor progression through the downregulation of transforming growth factor-β (TGF-β) and its partners and its relationship with the hypoxic state in the A172 human glioblastoma cell line, derived from the brain tissue of a 53-year-old patient.

## 2. Results

### 2.1. Multilayer Bioinformatics Analysis as an Identifier of Target Genes and Enriched Pathways Related to Immunometabolic Pathways

**Figure 1:**
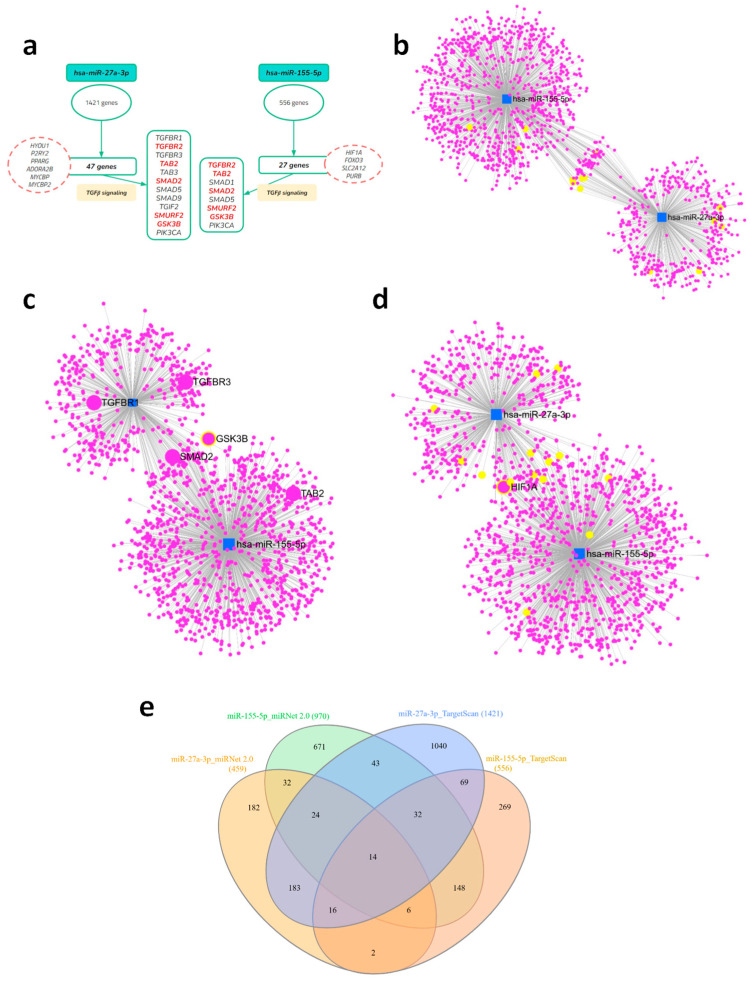
Multilayered analysis of the targets of the selected microRNAs, with emphasis on the TGF-β signaling pathway. (**a**) Initial target prediction by TargetScanHuman (release 8.0) identifying genes related to the TGF-β pathway, including receptors, SMADs, and modulators. Genes of interest in the pathway are highlighted in red. (**b**) Gene–miRNA interaction networks generated by miRNet for hsa-miR-27a-3p and hsa-miR-155-5p, highlighting the identified TGF-β pathway genes in yellow. (**c**,**d**) Combined target network of both miRNAs, showing overlap and shared interactions, highlighting target genes of TGF-β and HIF-1α signaling. (**e**) Venn diagram illustrating the intersection between predicted (TargetScan) and validated (miRNet) targets, highlighting common and unique genes for each miRNA.

Based on in silico screening and in vitro validation of differentially expressed miRNAs in glioblastoma, performed in our previous study [24], we identified miR-27a-3p and miR-155-5p as relevant targets in tumor progression. To deepen our understanding of the pathways regulated by these miRNAs, we performed a multilayered bioinformatic analysis. Based on these targets, we initially conducted a predictive analysis using TargetScan 8.0, which allowed us to map a broad set of potential target genes and identify enriched biological pathways associated with microRNAs, with emphasis on the TGF-β signaling pathway, including core components (TGF-β1, TGFBR1/2) and direct and indirect regulators of this cascade (Figure 1a). In this case, we found 1421 target genes for miR-27a-3p and 556 genes for miR-155-5p. Considering the metabolic and regulatory aspects of the pathways of interest, we limited the targets to 47 and 27 genes modulated, respectively, by miR-27a-3p and miR-155-5p (Figure 1a).

We then performed a complementary analysis using miRNet 2.0, a tool based on validated experimental data, which confirmed the association of miRNAs with the TGF-β pathway and demonstrated some overlap of target genes among the miRNAs in the study (Figure 1b). From the predictive and validated analyses, comprehensive lists of target genes and enriched pathways were obtained, which are fully presented in Supplementary Tables S1–S3. To visualize the relationship between the different sets, we constructed a Venn diagram that highlights the overlap and exclusivity of the genes identified by each approach (Figure 1e, Supplementary Table S3), revealing a core of genes that are recurrently present across multiple platforms. From the networks constructed with validated data, we observed a significant presence of genes related to TGF-β signaling, displayed here in yellow circles, especially in the context of miR-27a-3p. We also noted the presence of some targets already described in the predicted selection, which we highlighted as *SMAD2*, *GSK3B*, and *HIF1A*, shared as targets for both miRNAs (Figure 1c,d). This systematic approach consistently demonstrated the enrichment of immunometabolic pathways, with the TGF-β signaling pathway emerging as the most relevant and significant.

### 2.2. miR-27a-3p Downregulation Modulates Glucose Uptake and Metabolic Gene Expression, Whereas miR-155-5p Downregulation Regulates TGFB1 and HIF1A Expression

**Figure 2:**
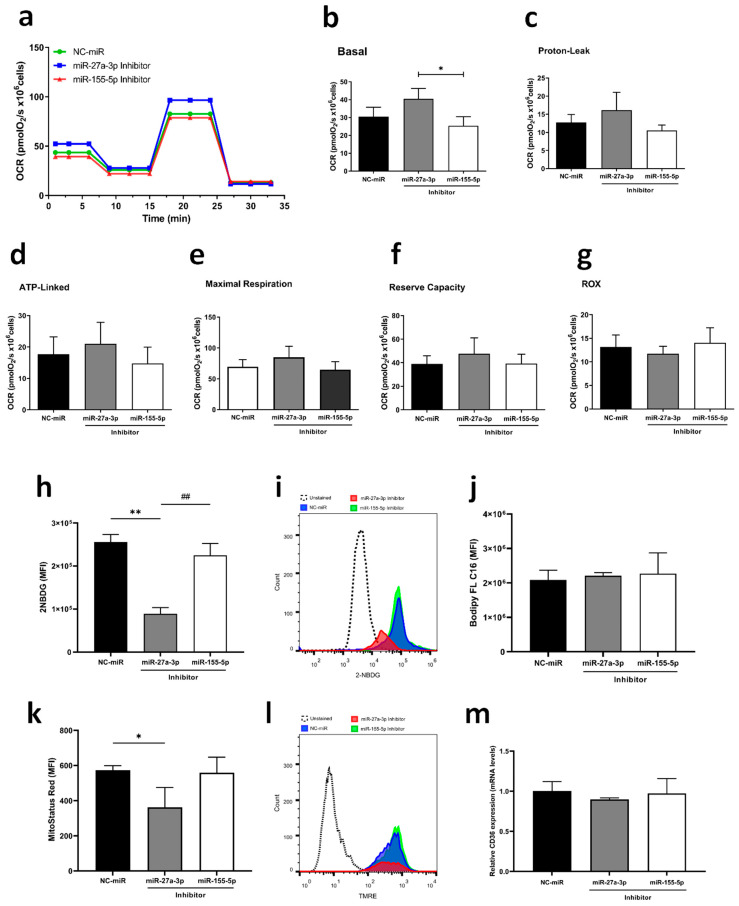
Inhibition of miR-27a-3p and miR-155-5p leads to changes in metabolic uptake and mitochondrial functionality. (**a**) Representative oxygen consumption rates (OCRs) measured by HRR. (**b**–**g**) Respiratory parameters assessed by HRR: Basal oxygen consumption (Basal); Oxygen consumption uncoupled to ATP synthesis (Proton Leak); Oxygen consumption coupled to ATP synthesis (ATP Linked); Maximum oxygen consumption (MAX); Reserve respiratory capacity (Reserve); Residual oxygen consumption (ROX). (**h**,**i**) Histogram and fluorescence intensities representing 2-NBDG uptake. (**j**) Green fluorescent fatty acid uptake, BODIPY FL C16. (**k**,**l**) Quantification of inner mitochondrial membrane depolarization levels by MitoStatus Red (TMRE) fluorescence assessed by flow cytometry in cells transfected with A172 and histogram representation; (**m**) fatty acid marker gene (CD36) expression in A172 cells assessed by RT-qPCR assay after transfection. miR-NC as negative control. Data are presented as mean ± SD of 3 independent experiments analyzed by ANOVA with post hoc Tukey correction. * *p* < 0.05, ** *p* < 0.01 vs. NC-miR, and ## *p* < 0.01 vs. miR-27a-3p inhibitor.

Regarding energy metabolism, a pathway that is greatly influenced during tumorigenesis and is related to TGF-β signaling, we assessed the impact of miR-27a-3p and miR-155-5p on oxygen consumption using High-Resolution Respirometry (HRR, Figure 2a–g), which only revealed that cells treated with miR-155-5p inhibitor had lower basal respiration when compared to miR-27a-3p inhibitor-treated cells (Figure 2b). However, when we measured glucose and fatty acid uptake using metabolic probes, the inhibition of miR-27a-3p reduced glucose uptake (*p* < 0.01) (Figure 2h,i) and mitochondrial membrane potential (*p* < 0.05) (Figure 2k,l) without any changes in fatty acids uptake and *CD36* expression (Figure 2j,m). In contrast, inhibition of miR-155-5p did not significantly affect glucose uptake, mitochondrial membrane potential, fatty acid uptake, or *CD36* expression compared to the NC-miR control, suggesting that metabolic regulation is more specifically mediated by miR-27a-3p.

**Figure 3:**
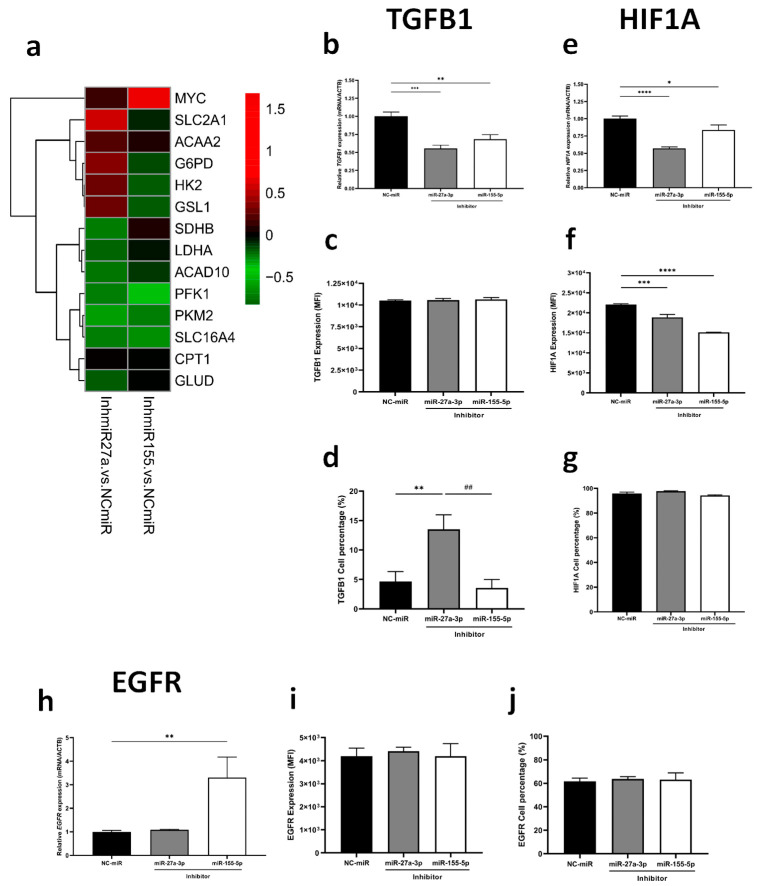
Inhibition of miRNAs modulates the expression of metabolic genes and affects gene and protein expression of key targets of tumorigenesis. (**a**) Heatmaps of relative expressions of metabolic genes assessed by RT-qPCR. (**b**–**d**) TGBF1, (**e**–**g**) HIF1A, and (**h**–**j**) EGFR gene and protein expression by RT-qPCR and flow cytometry, respectively. miR-NC as negative control. Data are presented as mean ± SD of 3 independent experiments. * *p* < 0.05, ** *p* < 0.01, *** *p* < 0.001, **** *p* < 0.0001 vs. NC-miR, and ## *p* < 0.01 vs. miR-27a-3p inhibitor.

Recognizing miR-27a-3p’s action on metabolic regulation, we examined the expression of key genes from glycolysis, fatty acid metabolism, and glutaminolysis. Inhibiting miR-27a-3p resulted in significant downregulation of *PFKM*, *PKM2*, *LDHA*, *SDHB*, *ACAD10*, and *GLUD*, while *SLC2A1*, *HK2*, *G6PD*, and *GLS1* were upregulated (Figure 3a, Supplementary Table S4). On the other hand, reducing miR-155-5p expression led to a significant reduction of *SLC2A1*, *PFKM*, *PKM2* and *HIF1A* (Figure 3a, Supplementary Table S4). Taking into account related pathways, we observed significant downregulation of *TGFB1* with the inhibition of both miRNAs, separately (Figure 3b). Specifically, inhibition of miR-27a-3p led to enrichment of TGFB1-expressing cells (Figure 3d) without fluorescence intensity alteration (Figure 3c). Additionally, both miRNAs reduced gene and protein expression of HIF1A, whereas only miR-155-5p increased *EGFR* gene expression (*p* < 0.01) (Figure 3e–j). These findings underscore the distinct yet complementary roles of miR-27a-3p in disrupting metabolic pathways and related signaling mechanisms critical for tumor progression. This positions miR-27a-3p as a promising target for therapeutic intervention in glioma treatment. Despite this difference, we acknowledge that EGFR’s role following miRNA inhibition was modest at the protein level and was only minimally investigated.

### 2.3. miR-27a-3p and miR-155-5p Regulate TGF-β Signaling by Modulating Downstream Partners

**Figure 4:**
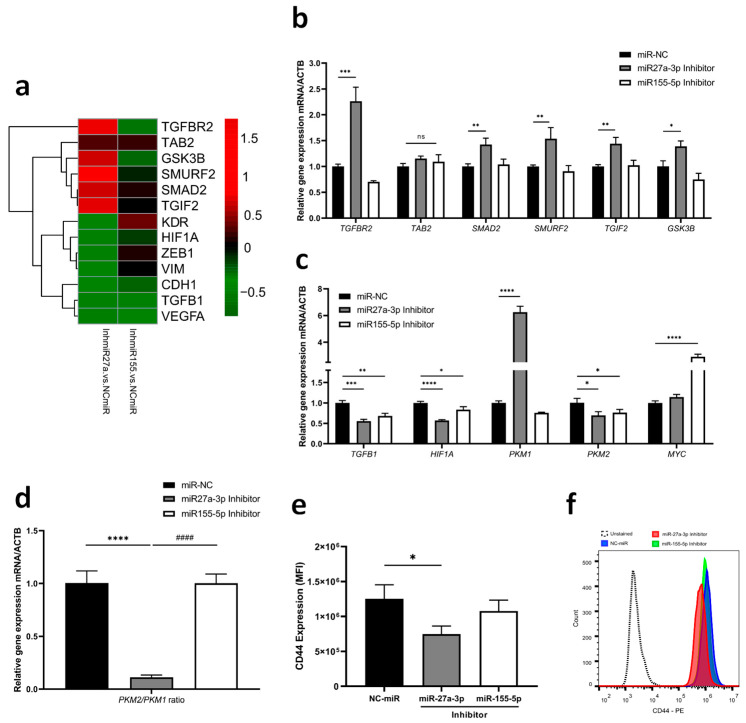
TGF-β signaling genes as targets of miR-27a-3p and miR-155-5p (**a**) Heatmap with qRT-PCR analysis was performed to determine the effects of inhibition on the predicted targets of TGF-β signaling, epithelial–mesenchymal transition, and angiogenesis. (**b**) Relative expression of shared target genes among study miRNAs after inhibition. (**c**) Relative expression of gene targets related to the functional duality of TGF-β. (**d**) Gene expression ratio of PKM isoforms. (**e**,**f**) Fluorescence intensities and representative histogram of CD44 protein expression. miR-NC as negative control. Data are presented as mean ± SD of 3 independent experiments. ns, not significant; * *p* < 0.05, ** *p* < 0.01, *** *p* < 0.001, **** *p* < 0.0001 vs. NC-miR, and #### *p* < 0.001 vs. miR-27a-3p inhibitor.

According to the bioinformatics analysis described before, many of these genes were closely related to the TGF-β signaling pathway, from which we selected six possible target genes for testing (*TGFBR2*, *TAB2*, *SMAD2*, *SMURF2*, *TGIF2*, *GSK3B*). Overall, miR-27a-3p inhibition in GBM cells resulted in a significant increase in the expression of certain genes, particularly some negative regulators of the TGF-β signaling pathway, such as *SMURF2*, *TGIF2,* and *GSK3B* (Figure 4a,b; Supplementary Table S4). On the other hand, miR-27a-3p inhibition leads to a reduced expression of genes related to epithelial–mesenchymal transition (EMT) and angiogenesis, such as *ZEB1, VIM,* and *VEGFA,* and its receptor (*KDR*) (Figure 4a). In addition to the reduction of *TGFB1* and *HIF1A,* inhibition of miR-27a-3p also reduced *PKM2* expression while increasing *PKM1* expression (Figure 4c). The pro-tumor regulatory effect exhibited by TGF-β in situations of hypoxia or advanced tumors may be regulated by the PKM isoforms. In our study, we observed that the *PKM2/PKM1* ratio was reduced when miR-27a-3p was inhibited (Figure 4d). Furthermore, inhibition of miR-27a-3p decreased CD44 protein expression, which is a relevant marker of TGF-β induction (Figure 4e,f). Regarding miR-155-5p inhibition, *MYC* expression was positively regulated (Figure 4c).

**Figure 5:**
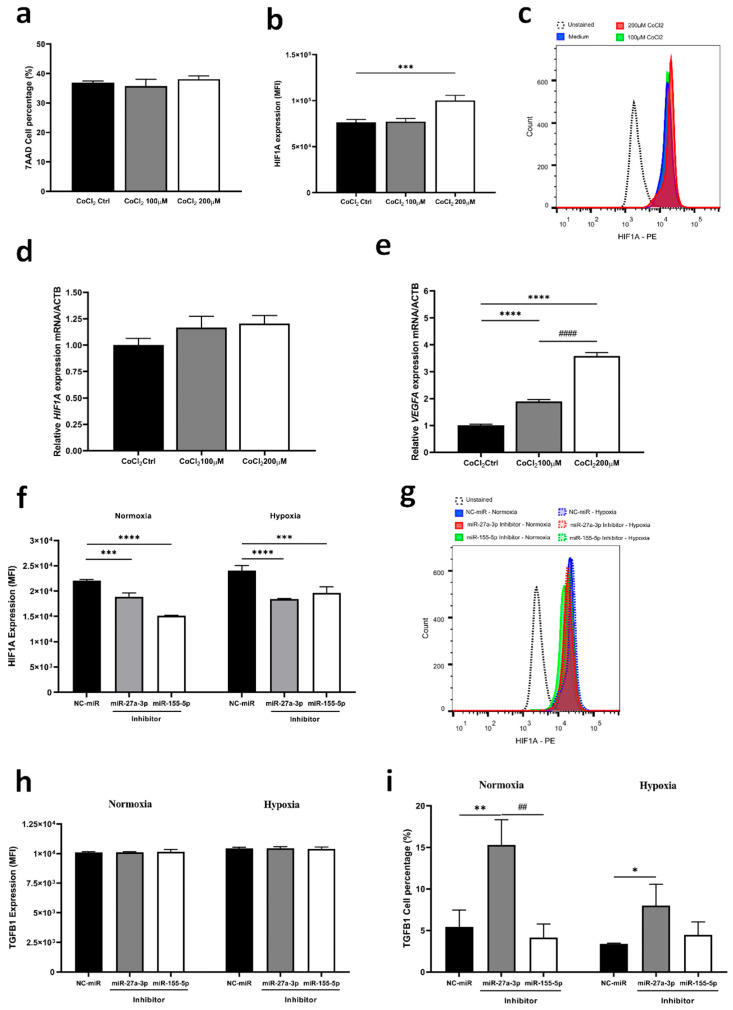
Inhibition of miR-27a-3p affects HIF1A expression under hypoxia and reduces TGF-β positive population. (**a**–**e**) Characterization of the gene and protein expression profile of HIF1A and the VEGFA gene under hypoxia induction protocol with CoCl_2_. (**f**,**g**) Protein expression by flow cytometry of HIF1A under normoxic and hypoxic conditions by induction with CoCl_2_. (**h**,**i**) Protein expression by flow cytometry of TGFB1 under normoxic and hypoxic conditions by induction with CoCl_2_. miR: microRNA. NC-miR: negative control. * *p* < 0.05, ** *p* < 0.01, *** *p* < 0.001, **** *p* < 0.0001 vs. NC-miR; ## *p* < 0.01 vs. miR-27a-3p inhibitor and #### *p* < 0.001 vs. CoCl_2_ 100 µM.

Given the significant roles of hypoxia-inducible factor 1 alpha (HIF-1α) and TGF-β under the hypoxic conditions of the tumor microenvironment, we induced hypoxia in tumor cells using cobalt chloride (CoCl_2_) with and without inhibition of miRNAs. Initially, we observed that the 200 µM CoCl_2_ concentration induced HIF1A protein expression and increased *VEGFA* gene expression (Figure 5a–e). Despite the hypoxic environment, both miRNA inhibitors reduced the expression of HIF1A in a similar manner compared to the control (Figure 5f,g). Interestingly, when comparing normoxia to hypoxia, it is clear that under the control condition and with inhibition of miR-155-5p, there was an increase in HIF1A (*p* < 0.028; *p* < 0.002, respectively), whereas the inhibition of miR-27a-3p did not alter the protein content (Figure 5f,g). Furthermore, we observed a significant reduction in cells expressing TGFB1 after miR-27a-3p inhibition in cells under hypoxia compared to normoxia (Figure 5h,i). Together, these findings highlight the distinct regulatory roles of miR-27a-3p and miR-155-5p in the TGF-β pathways and hypoxia, suggesting that inhibition of miR-27a-3p may modulate key regulatory players of the TGF-β pathway, even under hypoxic conditions, thus offering potential avenues for targeted therapy that affects the function and interaction of protumorigenic targets.

### 2.4. Combined Inhibition of TGF-β1 Receptor and miR-27a-3p Enhances Cytotoxic Effect in Glioblastoma Cells

**Figure 6:**
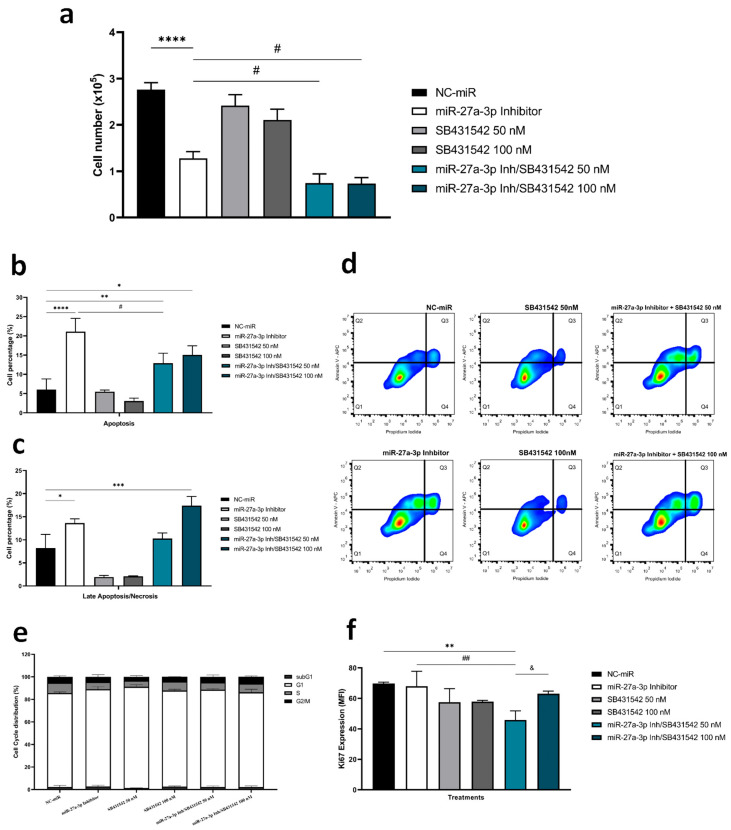
Co-treatment with specific TGF-β inhibitor and miR-27a-3p inhibitor further reduces cell viability. (**a**) Cell viability assessed by cell counts determined by staining with 0.4% trypan blue after inhibition of miR-27a-3p, SB431542 (50 and 100 nM) and inhibitor co-treatment. (**b**–**d**) Flow cytometry assay in A172 cells after treatment with miRNA and TGF-β inhibitors to quantify the ratio of apoptosis and late apoptosis/necrosis, with representative graphs of flow cytometry analysis for cell death. (**e**) Representative bar graphs of cell cycle progression by flow cytometry analysis for cells. (**f**) Proliferation rates measured by MFI for Ki-67. Data are presented as mean ± SD of 3 independent experiments. miR: microRNA. NC-miR: negative control. * *p* < 0.05, ** *p* < 0.01, *** *p* < 0.001, and **** *p* < 0.0001 vs. NC-miR; # *p* < 0.05, ## *p*< 0.01 vs. miR-27a-3p inhibitor, and ^&^ *p* < 0.05 vs. miR-27a-3p Inh/SB431542 50 nM.

Despite the observed impacts of miR-27a-3p inhibition, particularly in the TGF-β pathway, we noted an enrichment in TGFB1-positive cells post-treatment (Figure 3d and Figure 5i). Therefore, we applied a co-inhibition of miR-27a-3p and the TGF-β1 receptor (SB431542). The co-treatment induced more significant cell viability loss when compared to miR-27a-3p inhibition alone (Figure 6a), inducing early apoptosis and late apoptosis/necrosis (Figure 6b–d). We also observed a reduction in cell proliferation marker Ki67 after co-treatment compared to the control and the miRNA inhibitor alone (Figure 6f). However, there was no difference in the cell cycle progression (Figure 6e). These findings suggest that co-inhibition of miR-27a-3p and the TGF-β1 receptor induces cytotoxic effect leading to apoptosis in glioma cells, offering potential for improved intervention strategies.

We also observed that the combined inhibition of miR-27a-3p and TGF-β1 exerted additive or independent effects on TGF-β protein regulation, rather than a synergistic effect. This finding suggests that these microRNAs may modulate the TGF-β pathway through distinct or parallel mechanisms without substantial mutual amplification. Future studies employing synergy analyses are warranted to further clarify the nature of these interactions.

## 3. Discussion

Several studies have identified many miRNAs with potential roles in gliomas, and their differential expression patterns have been associated with GBM development and prognosis [12,25]. Therefore, this study examined the cellular and molecular effects of inhibiting two overexpressed miRNAs that play crucial roles in GBM progression. miR-27a-3p and miR-155-5p are highly expressed and identified as oncogenes in various cancers, including GBM [11,26,27,28]. Previous studies, including those published by our research group, have demonstrated that both miRNAs are positively regulated in different GBM cell lines and that their inhibition leads to a significant loss of viability, especially for miR-27a-3p [19,24,26,29,30,31]. In agreement, the increased expression of miR-27a-3p has been shown to affect glucose metabolism, especially by downregulating peroxisome proliferator-activated receptor gamma (PPARγ) [32]. In our findings, we observed a reduction in glucose uptake and mitochondrial membrane potential following miRNA-27a-3p inhibition, without affecting lipid uptake or *CD36* expression. To the best of our knowledge, this is the first time that miRNA-27a-3p has been shown to downregulate glucose intake in cancer, which could be directly related to the decreased viability of A172 GBM cells when this miRNA is inhibited. miR-27a-3p inhibition has also led to the negative regulation of glycolysis and TCA cycle genes (especially LDHA), reducing aerobic glycolysis potential. This process, also known as the Warburg effect, is a commonly taken route by rapidly growing cells [33,34]. Notably, three genes -*SLC2A1*, *HK2*, and *G6PD*- had their expression upregulated following miR-27a-3p inhibition as seen by Wai Hon et al. [35] and Kim et al. [36]. This may represent a compensatory mechanism in response to reduced glucose uptake and catabolism.

Brasciano et al. [20] and Quirico et al. [37] have demonstrated that miRNA-27a-3p influences oxidative phosphorylation and mitochondrial activity, promoting glycolysis or fatty acid metabolism, thereby favoring a more aggressive phenotype. On the other hand, there is an increase in the expression of *SLC2A1* (the gene that encodes the GLUT1 transporter) and *HK2* following the inhibition of miR-27a-3p, which may also be acting to compensate for the diminished glucose uptake observed. Furthermore, glucose can be directed to the pentose phosphate pathway (PPP), restoring NADPH levels. This can balance ROS levels and restore nucleotide levels for DNA synthesis and repair in extremely proliferative GBM cells [38,39]. The compensation mechanisms post-miR-27a-3p inhibition may be a possible survival strategy of A172 GBM cells.

Besides acting on glycolysis, Wai Hon et al. [35] observed that miR-27a facilitates mitochondrial activity and biogenesis. This activity appears to be regulated by the PPAR gamma-1α coactivator (PGC-1α), whose expression is modulated by miR-27a [20]. Furthermore, increased expression of miR-27a results in the elongation of mitochondria, an increase in mitochondrial membrane potential, and higher mitochondrial ATP levels, due to mitochondrial fusion [40]. Our results corroborate those of Tak et al. [40], who reported a reduction in mitochondrial membrane potential after miR-27a-3p inhibition, which is associated with mitochondrial fission and facilitates apoptosis [41,42]. Overall, the reduction in glucose uptake and the downregulation of the genes discussed in this study, following inhibition of miR-27a-3p, may lead to mitochondrial depolarization and trigger apoptosis.

High levels of TGF-β1 expression are associated with glioma malignancy and poor prognosis. TGF-β1 also acts on tumor cells’ metabolic reprogramming, favoring tumor progression [43,44,45,46]. This regulation occurs directly by modulating the expression of enzymes essential to metabolic processes and EMT [47,48]. TGF-β1-induced EMT activates several pathways, including the PI3K/Akt and MAPK pathways, which regulate glucose uptake and upregulate metabolic enzymes [49,50]. The increased expression of *HK2*, *PFKM*, *SLC2A1*, and *PKM2* induced by TGF-β has been observed in several types of cancer, including gliomas, particularly under hypoxic conditions [50,51,52,53]. This upregulation increases glucose uptake and upregulates the Warburg effect pathway [44,54,55]. Therefore, the decrease in metabolic gene expression mediated by anti-miR-27a-3p could be related to *TGFB1* downregulation, which weakens the tumor’s immunosuppressive profile and regulates key metabolic characteristics of cancer cells, ultimately leading to apoptosis.

TGF-β plays a paradoxical role in cancer, depending on microenvironmental conditions such as oxygen levels, and is closely related to its interaction with HIF-1α, which in turn regulates the expression of many glycolysis-related genes [56]. For instance, HIF-1α can alter the function of TGF-β in glucose metabolism through Smad3, providing environmental conditions that regulate the dual role of TGF-β in glucose metabolism under both normoxic and hypoxic conditions in the TME [50,57]. In addition, miRNAs play a crucial role in this regulatory axis by modulating both HIF-1α expression and TGF-β signaling. TGF-β, HIF-1α, and miRNAs form a complex feedback loop, where TGF-β can induce the expression of miRNAs that negatively regulate HIF-1α, while HIF-1α can influence TGF-β signaling and miRNA synthesis, creating a dynamic balance that contributes to cellular plasticity and tumor progression [58,59,60].

**Figure 7:**
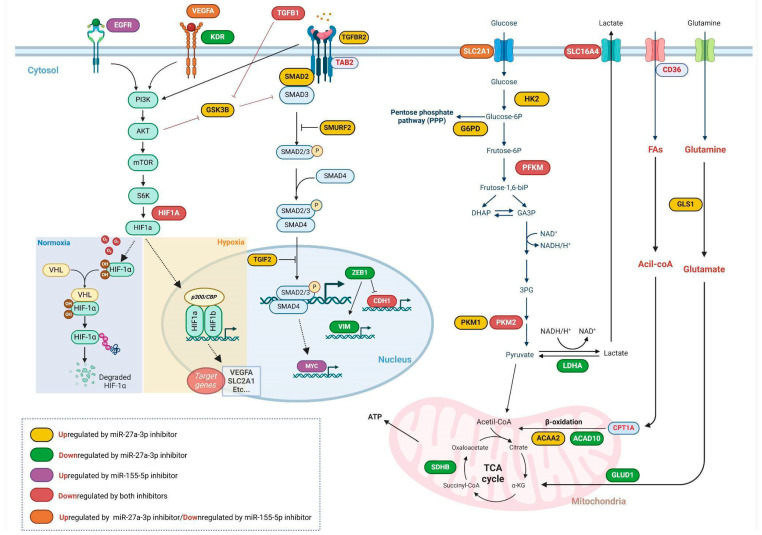
Modulation of gene expression following miR-27a-3p and/or miR-155-5p inhibition. This diagram provides a comprehensive overview of how different signaling and metabolic pathways are regulated within tumor cells, highlighting the influence of specific microRNA inhibitors such as miR-27a-3p and miR-155-5p under diverse conditions, illustrating the regulation of important receptors, such as EGFR and VEGFA, and crucial signaling pathways, including PI3K/AKT/mTOR and TGF-β, and their partners, as well as key metabolic pathways. Created in Biorender. Weber, A.F. (2025) https://app.biorender.com/t-68bd7d389179ec9ed84c16f8 (accessed on 4 September 2025). The figure was created with BioRender.com.

The axis of interaction between these targets is related to the modulation of SMAD partners, which leads to the activation of the TGF-β pathway and also selects the functional profile of the cytokines [50]. Furthermore, Kit et al. [60] demonstrated that the transcriptional activity of SMAD family genes is directly associated with tumor malignancy. Studies have reported that miR-27a and miR-155 target SMAD2 and SMAD4, which may lead to downregulation of TGF-β1 in some cancers, which is important for modulating the antitumor effects of the pathway in early-stage tumors [61,62]. Additionally, SMAD3, which activates the TGF-β pathway and can interact with HIF1A, contributes to a feedback loop that modulates the cellular response to TGF-β under hypoxic conditions [50]. Furthermore, miR-27a-3p and miR-155-5p are upregulated in response to TGF-β and HIF-1α and, in turn, regulate the expression of SMAD3, HIF-1α, and other components of the TGF-β signaling pathway. This interaction establishes an additional feedback mechanism that links the expression of TGF-β/HIF-1α and miR-27a-3p/miR-155-5p [59,63,64], as summarized in Figure 7.

The negative regulation of miRNAs in the study may act as a pivotal component in regulating the aforementioned targets. The inhibition of miR-27a-3p promotes increased expression of negative regulators of the TGF-β1 pathway, such as SMURF2 and GSK3B, which interact directly and indirectly with SMAD3 [65,66]. There is evidence that, in certain contexts, SMADs can collaborate with HIF1A to co-regulate the expression of target genes that are critical for the cellular response to the TME, which is often hypoxic [67]. Both TGF-β pathway inhibitors previously mentioned can be regulated by miRNAs, which adjust the intensity of TGF-β response, either by ubiquitination (Smurf2) or SMADs phosphorylation (GSK-3b) [68,69]. Inhibition of GSK3B can lead to sustained TGF-β1 signaling, promoting EMT, a process associated with tumor progression and metastasis [69]. This finding aligns with our results, as miR-27a-3p inhibition not only increased *GSK3B* expression but also negatively modulated genes associated with the mesenchymal phenotype in EMT, such as *ZEB1* and *VIM*.

CD44 is a cell adhesion molecule that promotes cancer cell proliferation and metastasis by upregulating TGF-β expression [70]. Studies highlight the importance of CD44, miRNA, and TGF-β signaling in tumor progression in glioma [71,72]. CD44 also plays a downstream role in TGF-β signaling by regulating EMT transcription factors, including Snail, Zeb1, and vimentin, which are linked to poor prognosis, particularly in mesenchymal GBM [72,73]. Our findings corroborate the existing literature, demonstrating that miR-27a-3p inhibition affects CD44 expression, which is possibly related to the reduction of *ZEB1* and *VIM*, leading to EMT.

Studies have revealed the functional duality of TGF-β and its interaction with PKM isoforms, hypoxic environments, and metabolic reprogramming [50]. Furthermore, the interaction between SMADs’ partners and HIF-1α favors metabolic reprogramming and tumor progression by benefiting PKM2 expression, an isoform prevalent in tumor cells, providing proliferating cells with differential control over glycolytic flux [74]. Our study demonstrated a reduction in the *PKM2/PKM1* ratio with miRNA-27a-3p inhibition, favoring PKM1 isoform. PKM1 is more prevalent in low-grade gliomas, which are subtypes with better prognosis [74,75]. Moreover, it has been observed that tumors with molecular subtypes associated with a worse prognosis exhibit higher expression of TGF-β1 and SMAD3. Together with HIF-1α, these factors promote the PKM2 isoform, which is linked to a less favorable prognosis profile [50,76].

Our findings demonstrate that inhibition of miRNA-27a-3p reduces the gene and protein expression of HIF-1α, which is sustained in the presence of hypoxia inducers. The interaction between these pathways not only amplifies cellular responses to hypoxia but can also direct cellular behavior towards more aggressive states, especially in diseases such as cancer. A detailed understanding of these interactions offers potential targets for therapeutic interventions in pathologies where TGF-β1 and HIF-1α signaling play critical roles.

Although our findings provide valuable insights into the role of miR-27a-3p and miR-155-5p in glioblastoma cells, we acknowledge that the use of a single cell line, A172, represents a limitation of this study. Glioblastoma is characterized by significant cellular and molecular heterogeneity, and validation of our results across multiple GBM cell lines would be preferable. Nevertheless, the selection of A172 was guided by our prior in vitro screening studies, which confirmed high expression levels of the miRNAs of interest, rendering it a relevant model for subsequent functional analyses [24]. Future studies should expand these investigations to additional glioblastoma cell lines to assess their response to miRNA inhibition, which will be critical for generalizing our findings.

To address this limitation, our future research plan includes the following: (i) validation of miR-27a-3p and miR-155-5p expression and functional effects across a broader panel of glioblastoma cell lines with distinct genetic and molecular backgrounds, including both IDH-mutant and IDH-wildtype subtypes; (ii) use of patient-derived primary cultures to capture inter-patient heterogeneity; (iii) application of functional assays with inhibitors or silencing strategies to provide proof of concept for the key targets identified; (iv) in vivo studies to evaluate the therapeutic potential of modulating these miRNAs. These steps will be essential to determine whether the regulatory mechanisms described here are broadly conserved or context-dependent, thereby enhancing the translational relevance of our findings.

## 4. Materials and Methods

### 4.1. Cell Culture and Cell Transfection

The human glioblastoma cell line derived from the brain tissue of a 53-year-old male as a model, A172 (RRID:CVCL_0131), was maintained in Dulbecco’s Modified Eagle Medium (DMEM, Gibco, Thermo Fisher Scientific, Waltham, MA, USA) supplemented with 10% fetal bovine serum (FBS, Gibco, Thermo Fisher Scientific, Waltham, MA, USA) and 100 U/mL penicillin/streptomycin maintained at 37 °C in a humidified atmosphere with 5% CO_2_. A172 cells were transiently transfected with target-specific miRNA inhibitors—mirVana miRNA inhibitor, negative control #1 (Invitrogen; cat. no. 4464076), mirVana miR-27a-3p inhibitor (Invitrogen; cat. no. 4464084, assay ID MH10939), or mirVana miR-155-5p inhibitor (Invitrogen; cat no. 4464084, assay ID MH28440)—at final concentrations of 50 nM, which was performed with Lipofectamine 2000 transfection reagent (Invitrogen, Carlsbad, CA, USA) for 6 h in Opti-MEM™ I Reduced-Serum Medium (Gibco). DMEM 10% SFB medium without antibiotics was replaced, and cells were incubated for 48 h until the start of experiments. Transfection efficiency was assessed through relative expression with qPCR.

### 4.2. Flow Cytometry

For the determination of the TGFB1, HIF1A, EGFR, and CD44 protein expression, after 48 h of transfection, cells were trypsinized, washed with 2% FBS, and centrifuged twice at 400× *g* for 6 min. Next, cells were labeled with the monoclonal antibodies, anti-EGF receptor (clone: EGFR.1; cat. 555997)/anti-CD44 (clone: 515; cat. 550989), from BD Biosciences, for 30 min and then washed twice. A fixation/permeabilization step was performed, followed by incubation for 30 min in the dark for anti-HIF-1 alpha (clone: Mgc3; cat. 12-7528-82) from Invitrogen and anti-TGFB1 (clone: TW4-2F8; cat. 562260) from BD Biosciences. Data was acquired by flow cytometry (FACSCanto II and BD Accuri cytometer, BD Biosciences, San Jose, CA, USA). The results were analyzed using FlowJo^®^ v10 software (FlowJo LLC, Ashland, OR, USA).

To assess the role of miRNAs in metabolic pathways, we checked glucose and fatty acid uptake and measured mitochondrial transmembrane potential. Cells were stained after 48 h transfection with metabolic probes such as 2-NBDG (Invitrogen™, Carlsbad, CA, USA; cat. N13195) at 40 μM for 20 min of incubation, BODIPY™ FL C16 (Invitrogen™, Carlsbad, CA, USA; cat. D3821) labeled at 2 µM for 30 min and MitoStatus TMRE (BD Biosciences, NJ, USA; cat. 564696) was performed for 15 min at 37 °C at a concentration of 100 nM. For the acquisition of samples and analysis of the results, the BD Accuri™ cytometer with the C6 Plus software (version 1.0.23.1; BD Biosciences, San Jose, CA, USA) was used.

Cell proliferation was assessed by Ki-67 staining. Briefly, after 48 h of transfection with miRNA inhibitors and treatments alone and combined with a specific TGF-β inhibitor (SB-431542, Sigma-Aldrich, St. Louis, MO, USA; cat. S4317), cells were suspended in BD Cytofix/Cytoperm^TM^ solution from BD Biosciences and incubated for 20 min on ice. Afterwards, the cells were washed with BD Perm/Wash^TM^ buffer (1X) and incubated with anti-Ki-67 (clone SolA15, 1:200; eBioscience, San Diego, CA, USA; cat. 11-5698-82) for 30 min on ice and then analyzed by flow cytometry (FACSCalibur, San Jose, California, USA, BD Biosciences). The results were analyzed using the FlowJo^®^ v10 software.

For cell cycle analyses, A172 cells were transfected and treated with a specific TGF-β inhibitor (SB431542), and after 48 h, the medium and cells were centrifuged at 400× *g* for 6 min. Afterwards, cells were washed once with phosphate-buffered saline (PBS; pH 7.4), centrifuged, and suspended in 300 µL of staining solution (0.5 mM Tris-HCl; pH 7.6; 3.5 mM trisodium citrate, 0.1% NP 40 (*v*/*v*), 100 μg/mL RNase and 50 μg/mL PI) for 15 min in the dark. Data was collected by flow cytometry (FACSCalibur, San Jose, California, USA, BD Biosciences) and analyzed using FlowJo v10 software.

Apoptotic or necrotic cells were quantified using an Annexin V-APC and PI dual stain kit, according to the manufacturer’s instructions (BD Biosciences; cat. 556547). Cells were transfected with NC-miR, miR-27a-3p inhibitor and treated with two concentrations of TGF-β specific inhibitor, SB431542 (50 and 100 nM), alone and co-treated with miRNA inhibitor. After 48 h of treatment, the medium and cells were harvested and centrifuged at 400× *g* for 6 min. Cells were washed twice with 1X Annexin V Binding Buffer and suspended in a buffer containing APC-conjugated Annexin V and PI (0.15 µg/mL^−1^). samples were incubated for 15 min at room temperature in the dark. Data was collected by flow cytometry (Accuri C6, BD Biosciences) and analyzed using FlowJo v10 software. Cells were classified as follows: viable cells (Annexin V and PI negative; Q1), early apoptotic cells (Annexin V positive and PI negative; Q2), late apoptotic and necrotic cells (Annexin V and PI positive; Q3/Annexin V negative and PI positive; Q4).

### 4.3. Cell Viability Assay

For the cell viability assay, the GBM cells were seeded in 12-well plates (8.0 × 10^4^ cells/well), allowed to grow until semi-confluence and transiently transfected with miRNA inhibitors, and treated with 50 and 100 nM of SB431542, alone or in cotreatment, for 48 h. At the end of the treatment, GBM cells were washed twice with PBS (pH 7.4) and detached with 0.5% trypsin/EDTA solution. Then, Trypan Blue (0.4%) was added, and cells were immediately counted in a hemocytometer to determine the number of viable cells. In each experiment, cells were counted in triplicate.

### 4.4. Hypoxia Induction In Vitro

To mimic hypoxia, different CoCl_2_ concentrations are chosen and added to the medium based on other publications tested with glioblastoma cancer cells [77,78]. To achieve HIF-1α stabilization in high levels, 50 and 100 μM of CoCl_2_ were added, for 48 h, to the cell media. RT-qPCR was performed to quantitatively estimate the changes in *HIF1A* and *VEGFA* mRNA expression in CoCl_2_-treated A172 cells. Furthermore, the cell viability of this treatment was carried out with marking by 7-AAD Viability Staining Solution (eBioscience™, BD Biosciences; cat. 00-6993-50) and analyzed by flow cytometry.

### 4.5. High-Resolution Respirometry (HRR) Assay

Oxygen consumption rate (OCR) was measured by high-resolution respirometry using Oroboros Oxygraph-2k (Oroboros Instruments, Innsbruck, Austria), and according to Gnaiger (2020) [79]. Measurements were performed in culture medium without SFB and data was acquired in pmol of O_2_ per second per million cells. Basal (routine) respiration was determined during oxygen consumption stabilization under normal conditions. Then, the ATP-synthase inhibitor oligomycin (2 μg/mL, Sigma-Aldrich, St. Louis, MO, USA) was added to assess ATP-linked and proton-leak respiration. Afterwards, maximum respiratory capacity was achieved by titration of mitochondrial uncoupler FCCP (100 nM, Sigma-Aldrich, St. Louis, MO, USA). Lastly, antimycin A (2.5 µM, Sigma-Aldrich) and rotenone (0.5 µM, Sigma-Aldrich, St. Louis, MO, USA) were added to obtain the residual oxygen consumption (ROX). The values obtained for each parameter were normalized by ROX. The parameters ATP-linked respiration and reserve capacity were calculated from the difference between basal and proton leak and the difference between maximum respiratory capacity and basal respiration, respectively. The DatLab software (version 7.4; Oroboros Instruments, Innsbruck, Austria) was used for data acquisition and analysis. The analyses were performed in quadruplicate. Figure 2a displays a representative curve of the experiment, while Figure 2b–g shows each parameter (values obtained from the curve) separately with statistical analysis through ANOVA analysis with Tukey post hoc correction.

### 4.6. Quantitative Real-Time Polymerase Chain Reaction (qPCR)

Total RNA content from transiently transfected cells was isolated using the TRIzol reagent (Invitrogen, Carlsbad, CA, USA) and was quantified by spectrophotometry. cDNA was synthesized from 2 µg of total RNA using M-MLV Reverse Transcriptase (Promega Corporation, Fitchburg, WI, USA), according to the manufacturer’s protocol. Real-time PCR was performed in triplicate on a StepOnePlus™ Real-time PCR system (Applied Biosystems, Waltham, MA, USA) using the GoTaq^®^ qPCR Master Mix (Promega Corporation, Fitchburg, WI, USA), also following the manufacturer’s instructions. The thermal cycling profile for gene expression consisted of an initial denaturation step at 94 °C for 2 min followed by 40 cycles of 15 s at 94 °C, 15 s at 60 °C, and 1 min at 72 °C for data acquisition. All primer sequences used in this study are described in Supplementary Table S5. Values were normalized to the relative expression levels of the housekeeping gene β-Actin (ACTB). To ensure the selection of the most accurate and stable reference gene for this analysis, the web-based tool RefFinder (https://www.ciidirsinaloa.com.mx/RefFinder-master/ (accessed on 15 July 2022)) was used [80]. Relative quantification was calculated using the ddCq method [81]. R package pheatmap (version 1.0.12; RRID:SCR_016418) was used to represent gene expression data in heatmaps.

### 4.7. Integrated Bioinformatics Analysis

To identify potential target genes regulated by the microRNAs of interest, we utilized the TargetScan 8.0 database (www.targetscan.org (accessed on 1 November 2023)) [82], a widely used bioinformatics tool that predicts microRNA-mRNA interactions based on seed sequence complementarity, evolutionary conservation, and site context features. In our study, we applied TargetScan to perform an initial in silico screening to identify putative targets for miR-27a-3p and miR-155-5p, focusing on genes involved in pathways relevant to glioblastoma pathogenesis, such as TGF-β signaling and hypoxia response. The predicted targets were subsequently filtered based on context and conserved site presence to increase prediction accuracy. This bioinformatics approach guided the selection of key genes for downstream experimental validation, enabling a more focused investigation of the molecular mechanisms mediated by these microRNAs.

Following the initial in silico screening with TargetScan, we performed additional bioinformatic analyses to investigate the functional associations of selected microRNAs and target genes. The online platform miRNet 2.0 was employed (https://www.mirnet.ca/miRNet/home.xhtml (accessed on 4 August 2025)) [83,84]. Initially, the miRNAs hsa-miR-27a-3p and hsa-miR-155-5p were input into the miRNAs module to generate an interaction network by mapping them to experimentally validated targets from the integrated knowledgebase miRTarBase. The resulting network was visualized using miRNet’s built-in graph layout to highlight relevant molecular interactions [83,84]. Subsequently, pathway enrichment analysis was performed within the platform using KEGG database to identify biological pathways significantly associated with the target genes regulated by the selected miRNAs. Enrichment significance was computed through hypergeometric testing, with multiple testing corrections applied to control the false discovery rate (FDR). This integrative approach enhanced the robustness of our target selection and informed subsequent functional validation experiments.

### 4.8. Statistical Analyses

Statistical analyses were conducted with GraphPad Prism 8.4.2 software^®^. Data is expressed as the mean ± S.D. The Shapiro–Wilk test was performed to verify if data was normally distributed. The data was compared among groups using one-way ANOVA, followed by Tukey’s post hoc test or Student’s *t*-test. Differences were considered significant when *p* < 0.05.

## 5. Conclusions

In conclusion, our study reveals that inhibiting miR-27a-3p and miR-155-5p significantly modulates TGF-β signaling, influencing its dual role in regulating metabolic reprogramming and cell death in A172 cells. Specifically, the inhibition of miR-27a-3p results in a reduction in glucose uptake and mitochondrial function, promoting apoptosis and shifting the metabolic profile toward a less aggressive phenotype. These findings underscore the intricate relationship between miRNAs, TGF-β signaling, and the tumor microenvironment (Figure 7). The present work highlights the potential of targeting miR-27a-3p to disrupt A172 cells, which may be the basis for identifying new therapeutic targets and strategies to modulate the survival mechanisms of tumor cells through post-transcriptional regulations and key pathways, like TGF-β signaling. Further studies are necessary, particularly with other GBM cell lines and murine models, to validate these findings and gain a deeper understanding of the roles of miR-27a-3p and miR-155-5p in glioblastoma progression, taking into account the known tumor heterogeneity.

## Data Availability

Data is contained within the article or Supplementary Material.

## Supplementary Materials

The following supporting information can be downloaded at: https://www.mdpi.com/article/10.3390/ijms26178729/s1.

## Abbreviations

CoCl_2_, cobalt chloride; DMEM, Dulbecco’s Modified Eagle Medium; EGFR, epidermal growth factor receptor; EMT, epithelial–mesenchymal transition; FBS, fetal bovine serum; GBM, glioblastoma; HIF-1α, hypoxia inducible factor 1 alpha; HRR, high-resolution respirometry; miRNAS, MicroRNAs; OCR, oxygen consumption rate; oncomiR, miRNA oncogenic; PGC-1α, PPAR gamma-1α coactivator; PPARγ, peroxisome proliferator-activated receptor gamma; qPCR, quantitative real-time polymerase chain reaction; ROX, residual oxygen consumption; TGF-β, transforming growth factor-β; TME, tumor microenvironment; UTRs, untranslated regions; WHO, World Health Organization.
