# Supplementary material for: Downregulation of miR-27a-3p Modulates TGF-β Signaling and Dysregulates Metabolism in Glioblastoma
Source: Int J Mol Sci. 2025 Sep 8;26(17):8729. doi: 10.3390/ijms26178729 (PMC12429544; doi:10.3390/ijms26178729)
Supplement: Supplementary file 1 [file ijms-26-08729-s001.zip › Supplementary table 4.pdf]

**Supplementary table 4**. Relative Gene Expression Analysis: Significance P-Values

| Gene ID | InhmiR27a vs. NCmiR | InhmiR155 vs. NCmiR |
|---|---|---|
| *ACAA2* | 0,0024 | 0,3701 (ns) |
| *ACAD10* | 0,0002 | 0,3053 (ns) |
| *CD36* | 0,537 (ns) | 0,9529 (ns) |
| *CDH1* | 0,0009 | <0,0001 |
| *CPT1A* | >0,9999 (ns) | >0,9999 (ns) |
| *EGFR* | 0,9735 (ns) | 0,0032 |
| *G6PD* | 0,0034 | 0,1279 (ns) |
| *GLS1* | 0,0258 | 0,16 (ns) |
| *GLUD1* | 0,0009 | 0,6751 (ns) |
| *GSK3B* | 0,0128 | 0,0674 (ns) |
| *HIF1A* | <0,0001 | 0,0154 |
| *HK2* | 0,0447 | 0,2287 (ns) |
| *KDR* | 0,0129 | 0,1739 (ns) |
| *LDHA* | <0,0001 | 0,5299 (ns) |
| *MYC* | 0,3555 (ns) | <0,0001 |
| *PFKM* | 0,0193 | <0,0001 |
| *PKM1* | <0,0001 | 0,5276 (ns) |
| *PKM2* | 0,0167 | 0,0471 |
| *SDHB* | <0,0001 | 0,2405 (ns) |
| *SLC2A1* | <0,0001 | 0,0277 |
| *SLC16A4* | <0,0001 | <0,0001 |

| | | |
|---|---|---|
| *SMAD2* | 0,0043 | 0,8836 (ns) |
| *SMURF2* | 0,0078 | 0,7013 (ns) |
| *TAB2* | 0,1762 (ns) | 0,4706 (ns) |
| *TGFB1* | 0,0002 | 0,0011 |
| *TGFBR2* | 0,0002 | 0,1406 (ns) |
| *TGIF2* | 0,0027 | 0,956 (ns) |
| *VEGFA* | 0,0081 | 0,014 |
| *VIM* | <0,0001 | 0,9207 (ns) |
| *ZEB1* | 0,0009 | 0,9038 (ns) |

(ns): not statistically significant
