# Supplementary material for: Downregulation of miR-27a-3p Modulates TGF-β Signaling and Dysregulates Metabolism in Glioblastoma
Source: Int J Mol Sci. 2025 Sep 8;26(17):8729. doi: 10.3390/ijms26178729 (PMC12429544; doi:10.3390/ijms26178729)
Supplement: Supplementary file 1 [file ijms-26-08729-s001.zip › Supplementary table 5.pdf]

Supplementary table 5. Primer sequences for mRNA targets evaluated by qPCR

Gene ID	mRNA Accession	Sequence of primer
ACAA2	NM_006111.3	*Forward* 5'- CTGCTCCGAGGTGTGTTTGTA-3'<br>*Reverse* 5'- GGCAGCAAATTCAGACAAGTCA-3'
ACAD10	NM_025247.6	*Forward* 5'- CAAACACTCGGCCTGTGAAAA-3'<br>*Reverse* 5'- ACTAGATCACGATTAGCCAGCC-3'
ACTB	NM_001101.5	*Forward* 5'- CCTGGCACCCAGCACAAT-3'<br>*Reverse* 5'- GACTCGTCATACTCCTGCTTG-3'
CD36	NM_001001547.3	*Forward* 5'- GTGGCAGCTGCATCCCATA-3'<br>*Reverse* 5'- CAGGGTACGGAACCAAACTCA-3'
CDH1	NM_001317185.2	*Forward 5'-* TGCCCAGAAAATGAAAAAGG-*3'*<br>*Reverse 5'-* GTGTATGTGGCAATGCGTTC-*3'*
CPT1A	NM_001876.4	*Forward* 5'- ATCAATCGGACTCTGGAAACGG-3'<br>*Reverse* 5'- TCAGGGAGTAGCGCATGGT-3'
EGFR	NM_201283.2	*Forward* 5'- CAGTGGCGGGACATAGTCAG-3'<br>*Reverse* 5'- CCCATTGGGACAGCTTGGAT-*3'*
G6PD	NM_001360016.2	*Forward* 5'- ACGACGAAGCGCAGACAG-3'<br>*Reverse* 5'- TCCGACTGATGGAAGGCATC-3'
GLS1	NM_014905.5	*Forward 5'-* TCTACAGGATTGCGAACGTCT-*3'*<br>*Reverse 5'-* CTTTGTCTAGCATGACACCATCT-*3'*
GLUD1	NM_001318906.2	*Forward* 5'- GACATCGTGCACTCTGGCT-3'<br>*Reverse* 5'- AGGTCACACCAGCTTCATTGT-3'
GSK3B	NM_002093.4	*Forward 5'-* CAACTGCCCGACTAACACCA-*3'*<br>*Reverse 5'-* TGAATCCGAGCATGAGGAGG-*3'*
HIF1A	NM_181054.3	*Forward* 5'- GACCGATTCACCATGGAGGG-3'<br>*Reverse* 5'- GTGGCAACTGATGAGCAAGC-3'
HK2	NM_001371525.1	*Forward* 5'- CCCCTGCCACCAGACTAAAC-3'<br>*Reverse* 5'- CAAAGTCCCCTCTCCTCTGG-3'
KDR	NM_002253.4	*Forward 5'-* CAAGTGGCTAAGGGCATGGA-*3'*<br>*Reverse 5'-* ATTTCAAAGGGAGGCGAGCA-*3'*
LDHA	NM_001135239.2	*Forward* 5'- ATGGCAACTCTAAAGGATCAGC-3'<br>*Reverse* 5'- CCAACCCCAACAACTGTAATCT-3'
MYC	NM_002467.6	*Forward* 5'- GGACCCGCTTCTCTGAAAGG-3'<br>*Reverse* 5'- TAACGTTGAGGGGCATCGTC-3'
PFKM	NM_001354742.2	*Forward* 5'- TGGGACTAAAAGGACTCTACCC-3'<br>*Reverse* 5'- CCCTGTGTAAGCCTCAAAGC-3'

PKM1	NM_001206798.3	*Forward* 5'- TCACTCCACAGACCTCATGG-3'<br>*Reverse* 5'- GAAGATGCCACGGTACAGGT-3'
PKM2	NM_001206798.3	*Forward* 5'- ATCGGTCCTCACCAAGTCTGG-3'<br>*Reverse* 5'- GAAGATGCCACGGTACAGGT-3'
SDHB	NM_003000.3	*Forward* 5'- GTGGCCCCATGGTATTGGAT-3'<br>*Reverse* 5'- CGGGTGCAAGCTAGAGTGTT-3'
SLC2A1	NM_006516.4	*Forward* 5'- GAACTCTTCAGCCAGGGTCC-3'<br>*Reverse* 5'- ACCACACAGTTGCTCCACAT-3'
SLC16A4	NM_001206951.2	*Forward* 5'- ACAGCCTGGATCTCCTCCAT-3'<br>*Reverse* 5'- ATGATGCTCCGGCAAAAGGA-3'
SMAD2	NM_001003652.4	*Forward 5'*- GAGCAGAATGGGCAGGAAGA-*3'*<br>*Reverse 5'*- AGAGCAAGTGCTTGGTATGG-*3'*
SMURF2	XM_054316942.1	*Forward 5'*- AACAAGACCGGCGTCAGAAT-*3'*<br>*Reverse 5'*- TGTTCATAGCCTTCGGGTAG-*3'*
TAB2	NM_001292034.3	*Forward 5'*- GCCCAAGGAAGCCACCAAAT-*3'*<br>*Reverse 5'*- AGAGAGAACAGCACAGCAGG-*3'*
TGFB1	NM_000660.7	*Forward* 5'- TCAAGCAGAGTACACACAGCA-3'<br>*Reverse* 5'- TGAGGTATCGCCAGGAATTGT-3'
TGFBR2	NM_001024847.3	*Forward 5'*- CTCCAGGCCCCCTCCTG-*3'*<br>*Reverse 5'*- CCGCTGCTCGTCATAGACC-*3'*
TGIF2	NM_001199513.2	*Forward 5'*- TCGCGCTCCGCACAAAG-*3'*<br>*Reverse 5'*- ATCTTCACCGACTCCTTGGG-*3'*
VEGFA	NM_001025366.3	*Forward 5'*- ACGAAAGCGCAAGAAATCCC-*3'*<br>*Reverse 5'*- CTCCAGGGCATTAGACAGCA-*3'*
VIM	NM_003380.5	*Forward 5'*- GAGAACTTTGCCGTTGAAGC-*3'*<br>*Reverse 5'*- GCTTCCTGTAGGTGGCAATC-*3'*
ZEB1	NM_001174095.2	*Forward 5'*- GGCGCAATAACGGAAAGGAAGG-*3'*<br>*Reverse 5'*- GACAGCAGTGTCTTGTTGTTGT-*3'*
